# Imported malaria in pregnancy in Madrid

**DOI:** 10.1186/1475-2875-11-112

**Published:** 2012-04-11

**Authors:** Beatriz C Jiménez, Pedro Cuadros-Tito, Jose M Ruiz-Giardin, Gerardo Rojo-Marcos, Juan Cuadros-González, Eduardo Canalejo, Noemi Cabello, Juan V San Martín, Ana M Barrios, Juan Hinojosa, Laura Molina

**Affiliations:** 1 Internal Medicine Department, University Hospital Fuenlabrada, Madrid, Spain; 2 Internal Medicine Department, University Hospital Príncipe de Asturias, Alcalá de Henares, Madrid, Spain; 3 Clinical Microbiology and Parasitology Department, University Hospital Príncipe de Asturias, Alcalá de Henares, Madrid, Spain; 4 Clinical Laboratory Department, Microbiology Unit, University Hospital Fuenlabrada, Madrid, Spain

**Keywords:** Malaria, Imported, Pregnancy, Immigration

## Abstract

**Background:**

Malaria in pregnancy is associated with maternal and foetal morbidity and mortality in endemic areas, but information on imported cases to non-endemic areas is scarce.

The aim of this study was to describe the clinical and epidemiological characteristics of malaria in pregnancy in two general hospitals in Madrid, Spain.

**Methods:**

Retrospective descriptive study of laboratory-confirmed malaria in pregnant women at the Fuenlabrada University Hospital and the Príncipe de Asturias University Hospital, in Madrid, over a six- and 11-year period, respectively. Relevant epidemiological, clinical and laboratory data was obtained from medical records.

**Results:**

There were 19 pregnant women among 346 malaria cases (5.4%). The average age was 27 years. The gestational age (trimester) was: 53% 3^rd^, 31% 1st, 16% 2^nd^. All but one were multigravidae. Three were HIV positive. All were sub-Saharan immigrants: two were recently arrived immigrants and seventeen (89%) had visited friends and relatives. None had taken prophylaxis nor seeked pre-travel advice. Presentation: 16 symptomatic patients (fever in fourteen, asthenia in two), three asymptomatic. Median delay in diagnosis: 7.5 days. Laboratory tests: anaemia (cut off Hb level 11 g/dl) 78.9% (mild 31.6%, moderate 31.6%, severe 15.8%) thrombocytopaenia 73.7%, hypoglycaemia 10.5%. All cases were due to *Plasmodium falciparum*, one case of hyperparasitaemia. Quinine + clindamycin prescribed in 84%. Outcomes: no severe maternal complications or deaths, two abortions, fifteen term pregnancies, no low-birth-weight newborns, two patients were lost to follow-up.

**Conclusions:**

Though cases of malaria in pregnancy are uncommon, a most at risk group is clearly defined: young sub-Saharan mothers visiting friends and relatives without pre-travel counselling and recently-arrived immigrants. The most common adverse maternal and foetal effects were anaemia and stillbirth. Given that presentation can be asymptomatic, malaria should always be considered in patients with unexplained anaemia arriving from endemic areas. These findings could help Maternal Health programme planners and implementers to target preventive interventions in the immigrant population and should create awareness among clinicians.

## Background

Malaria in pregnancy is known to be a primary cause of morbidity and mortality in both mother and foetus in endemic areas. It is in this setting that this form of malaria is being extensively studied and where current knowledge derives from. Information on the incidence and relevance of this particular type of malaria in non-endemic, industrialized areas is scarce [1-3]. Though malaria vanquished from Spain in 1964, arrival of migrants from endemic areas and increased international tourism during the past 2 decades has led to a reemergence of this parasitic disease as an imported infection. In fact, falciparum malaria is one of the most common potentially fatal tropical diseases that can be imported to Spain.

In Spain, around 350-400 cases of malaria/year have been reported in the past five years to the national communicable diseases database, though it is estimated that the total number of imported cases is over 400, because of under-reporting. The majority are due to *Plasmodium falciparum*. The region of Madrid registers a proportionally large number of cases: 21-37% of all malaria cases diagnosed in Spain during the period 2006-2009 were reported from Madrid. In 2009, the incidence rate (IR) was 2,0/100,000, three-times higher than the national IR [4].

Within the region of Madrid, a high proportion of cases are reported from the southern and eastern areas where a large number of migrants originally from endemic areas have settled. The Fuenlabrada University Hospital (FUH) and the Príncipe de Asturias University Hospital (PAUH) are both located in these two particularly migrant populated areas. Nonetheless, as reported by the regional surveillance network, these hospitals diagnosed 53% of malaria cases in Madrid in both 2008 and 2009, despite only 10% of total regional population living in these areas [4].

Although imported malaria to Spain has already been described in several reports from different regions and health care settings and guidelines highlighting the risk and prevention strategies during pregnancy have been published, little is known about the relevance and characteristics of imported malaria during pregnancy [5,6].

The aim of this study was to describe the clinical and epidemiological characteristics and outcomes of malaria in pregnancy in these two general hospitals in a non-endemic country.

## Methods

A retrospective descriptive review was conducted on all pregnant women with laboratory confirmed malaria diagnosed in the PAUH and the FUH, during the period January 1^st ^2000 to June 1^st ^2011 and January 1^st ^2005 to June 1^st ^2011, respectively. Laboratory criteria used for confirmation of diagnosis was the demonstration of asexual forms of malaria parasites in a thick and/or thin blood film. Positive antigen detection (second generation test for rapid detection of *P. falciparum *antigen-PfHRP2) and positive polymerase chain reaction test for *Plasmodium *nucleic acid was considered additional support to diagnosis. Clinical, epidemiological and laboratory information was collected from medical records.

Demographic characteristics included age, country of origin, time elapsed since arrival to Spain, reason for travel to endemic area (migrant visiting friends and relatives-VFR- or national traveller) and recently arrived migrant status. Epidemiological data included: area where malaria was acquired from (last endemic area visited), month, incubation period (number of days elapsed between the last day in endemic area till symptom onset), delay in diagnosis (days from symptom onset till microbiological diagnosis) and written evidence of any pre-travel advice.

Clinical data collected included previous medical history (co-morbid conditions), current presentation classified as fever, flu-like, gastrointestinal or respiratory symptoms and other presentations, WHO criteria for severe malaria (WHO, 2006), any remarkable clinical sign observed at diagnosis and information on chemoprophylaxis (no/inadequately taken prophylaxis or adequate prophylaxis).

Laboratory data included anaemia assessment according to WHO standards (anaemia was considered present when haemoglobin (Hb) < 11 g/dl; cut off levels for mild, moderate and severe anaemia were Hb 9-10.9 g/dl, Hb7.1-8.9 g/dl and Hb < 7 g/dl, respectively), platelet count and classification as mild (100,000-150,000/mm^3^), moderate (80,000-100,000/mm^3^) and severe (< 80,000/mm^3^) thrombocytopenia and any other remarkable laboratory finding at diagnosis. Microbiological data included *Plasmodium *species, parasitaemia at diagnosis, and results of rapid diagnostic tests and PCR if they had been carried out. Information on the treatment prescribed and clinical and parasitological follow-up at days 3, 7 and 30 after treatment onset was also collected.

Obstetrical data collected included parity, gestational age at diagnosis, prior obstetrical classification as high-risk pregnancy and reason for the patient being classified as such, timing of delivery, pregnancy outcomes and newborn birth weight (low birth weight-LBW- defined as < 2500 g). Continuous variables are reported as mean +/- standard deviation (SD) or median and interquartile range (IR). Categorical variables are reported as frequencies and percentage of each category.

## Results

There were 19 malaria cases in pregnant women among 346 malaria cases diagnosed in both institutions during the study period (5.4%). Demographical, epidemiological, clinical, microbiological and laboratory characteristics are summarized in Table 1.

**Table 1:** Clinical and epidemiological characteristics and outcomes of 19 cases of imported malaria in pregnancy

Variables		Frequency	(%)
Country of Origin			
	
	Equatorial Guinea	11	(57.9)
	
	Nigeria	6	(31.6)
	
	Congo	1	(5.3)
	
	Ghana	1	(5.3)
	
Mean Age Years (+/- SD)		27	(+/-5.3)

Reason for Travel			
	
	VFR	17	(89.5)
	
	National Traveller	0	
	
Recent Immigration	Yes	2	(10.5)

Chemoprophylaxis			
	
	None/Incorrect/Incomplete	19	(100)

Co-morbidities			
	
	None	11	(57.9)
	
	HIV	3	(15.8)
	
	Hypertension	1	(5.3)
	
	Smoker	1	(5.3)
	
	Other	3	(15.8)

Gestational Age			
	
	Third Trimester	10	(52.6)
	
	Second Trimester	3	(15.8)
	
	First Trimester	6	(31.6)

Parity			
	
	Multigravidae	18	(94.7)
	
	Primigravidae	1	(5.3)
	

Classification As High Risk Pregnancy	Yes	6	(31.6)

Clinical Presentation			
	
	-Asymptomatic	3	(15.8)
	
	-Symptomatic	16	(84.2)
	
	Fever	14	(73.6)
	
	Fever Alone	7	(36.8)
	
	Fever + Gastrointestinal	4	(21.1)
	
	Fever + Flu-Like Symptoms	3	(15.8)
	
	Other	2	(10.5)

- Signs			
	
	None	17	(89.5)
	
	Splenomegaly (At examination and/or ultrasound)	1	(5.3)
	
	Hepatosplenomegaly (At examination and/or ultrasound)	1	(5.3)

Laboratory Findings			
	
	-Anaemia (Hb < 11 g/Dl)	15	(78.9)
	
	Mild (9-10.9 g/Dl)	6	(31.6)
	
	Moderate (7-8.9 G/Dl)	6	(31.6)
	
	Severe (Hb < 7 g/Dl)	3	(15.8)
	
	-Thrombocytopaenia (< 150,000/Mm^3^)	14	(73.7)
	
	Mild (100,000-150,000/Mm^3^)	8	(42.1)
	
	Moderate (80,000-10,000/Mm^3^)	2	(10.5)
	
	Severe (< 80,000/Mm^3^)	4	(21.1)
	
	-Hypoglycaemia (Serum Glucose < 60 Mg/Dl, With Or Without Symptoms)	2	(10.5)

Microbiology			
		
	- *P. falciparum *(One Mixed Infection)	19	(100)
	
	Parasitaemia > 4%	1	(5.3)
	
	Parasitaemia 1-4%	5	(26.3)
	
	Parasitaemia < 1%	11	(57.9)
	No data	2	(26.3)
	
Diagnostic delay days Median (IR)		7.5	(2.7-21.7)

Treatment			
	
	Quinine + Clindamycin	16	(84.2)
	
	Mefloquine	1	(5.3)
	
	Atovaquone + Proguanil*	2	(10.5)

Maternal outcome			
- Death		0	(0)
		
-Clinical complications		0	(0)

-Anaemia improvement (N = 15)	Yes	9	(60)
		
	No data	6	(40)

Foetal outcome			
	
	-Abortion	2	(10.5)
	
	-Delivery Term	15	(78.9)
	
	No data (Lost to follow-up)	2	(10.5)

-Birth weight N = 15	> 2,500 g	15	(100)
	
	< 2,500 g	0	(0)

Parasitological follow-up			
	
	Undetectable parasitaemia	12	(63.2)
	
	No data	7	(38.9)

VFR: visiting friends and relatives

SD: standard deviation

IR: interquartile range (25^th^-75^th ^percentile)

* Two patients received atovaquone-proguanil: one after premature abortion and another after term delivery

All but one were multigravidae. All patients were young sub-Saharan African women, mainly from Equatorial Guinea (58%). There were no national travellers. All but two (89%) were VFR. Median time living in Spain of the 15 VFR for whom this information was available was 6 years (IR 10 months-10 years). The two non-VFR patients were recently arrived immigrants to Spain, one month and six months before clinical presentation, respectively.

Malaria was acquired in their countries of origin in all cases except for one patient who had visited relatives in another endemic country. There was no written evidence of any pre-travel advice in any case and none had correctly taken chemoprophylaxis. Sixteen patients (83.5%) had acquired malaria travelling during either summer or Christmas holiday periods. No information was available on intermittent preventive treatment.

The majority of patients (58%) did not have any other medical condition prior to malaria. There were three HIV positive women: two CDC stage A2 and one A1. Six pregnancies (32%) had previously been classified as high risk. The reasons for this classification were: HIV positivity in three cases, hypertension in one case, gestational diabetes in another case and, finally, one patient was undergoing close follow-up because of a history of fever during her stay in Africa.

There were three asymptomatic cases. The predominant clinical syndrome among the symptomatic women was fever (14 patients) with or without associated symptoms. The 2 symptomatic non-febrile women referred asthenia as major complaint. Malaria parasites were detected in the three asymptomatic cases because of different diagnostic work-ups: in one case, the patient was being studied for unexplained 3^rd ^trimester severe anaemia, in one of the HIV positive patients both malaria and microfilariae were observed in the course of a routine 3^rd ^trimester blood test, the other HIV positive patient was found to have malaria after the finding of an unexplained hepatosplenomegaly during a routine obstetric ultrasound. Other than this patient and another patient with splenomegaly, clinical examination was unremarkable in all cases.

Date of symptom onset was recorded in 15 of the symptomatic cases. Symptom onset occurred while still in an endemic area in five cases (17 days to 1 day before returning to Spain). Nine patients started referring symptoms once in Spain, ranging from seven to 69 days after the last day in the endemic area (median 11 days; IR 3.5-36 days). Median delay in diagnosis was 7.5 days (IR 2.7-21.7 days). In 7 cases, diagnosis was made more than one week after symptom onset.

None of the patients presented clinical features for severe malaria and only in one case was a laboratory criteria observed, a parasitaemia level over 4%. The most frequent laboratory finding was anaemia, (79%), followed by thrombocytopaenia (74%) and hypoglycaemia (two cases, both asymptomatic and over 40 mg/dl, cut off level for severe malaria).

All cases were *P. falciparum *malaria, with one mixed infection. A rapid diagnostic test was used as complementary diagnostic support in 12 cases, and PCR testing was carried out in eight cases, yielding positive results for *P. falciparum *in all cases. Data on parasitological follow-up was incomplete. Twelve patients had confirmed negative parasitaemia at any time during follow-up.

The majority of cases (84%) were treated with quinine plus clindamycin. Patients with severe anaemia received blood transfusions. There were no maternal complications or deaths. Pregnancy outcomes were favourable in 15 cases: all term pregnancies, no low birth weight newborns. There were two premature abortions. Anaemia was reassessed in 10 patients; in all but one there was an increase in Hb levels after treatment.

## Discussion

This study describes the characteristics of 19 *P. falciparum *malaria cases among pregnant women attending the two suburban general hospitals where a large proportion of malaria cases are reported from in the region of Madrid. Results show that, though this presentation is uncommon, a most at risk group is clearly defined: young sub-Saharan mothers visiting friends and relatives without pre-travel counselling and recently arrived immigrants.

Pregnant women are at increased risk for malaria infection. Increased susceptibility has long been recognized and multiple factors have been identified, yet, there are still many gaps in knowledge that need to be addressed. Placental infected erythrocytes differ from those found in peripheral blood because they present surface antigens that act as ligands permitting adhesion to chondroitin-sulphate A receptors in the intervillous space. IgG antibodies against pregnancy specific parasites are gradually developed resulting in a unique form of immunity: parity-specific immunity.

Thus, in endemic areas, the clinical impact varies depending on transmission. In highly endemic areas with stable transmission, the frequency and severity of the infection is higher in primigravidae than multigravidae, while, in hypoendemic areas with unstable transmission, women have less opportunity to develop parity-specific immunity and so are equally susceptible across the parity.

Malaria is associated with maternal and foetal morbidity and mortality. Percentages of maternal deaths vary depending on studies: up to 23% or up to 17% in hospital-based and community-based studies in Africa, respectively [7]. The most deleterious effects are caused by *P. falciparum*. In areas of high transmission, severe anaemia is common and is associated with morbidity and mortality. Foetal disease presents as preterm delivery, stillbirth and a higher risk of LBW and consequently increased risk of death in infancy, whether or not maternal symptoms are detected during pregnancy [8]. Unfortunately, it is difficult to estimate the burden of disease in non-endemic areas since epidemiological and clinical data is scarce and based on isolated cases, small series or cases within large series [1-3].

In the United States, basic epidemiological data is available from the Centre for Disease Control surveillance summaries. For instance, in 2005, a total of 21 cases of malaria were reported among pregnant women, representing 4% of cases among women. Among these, 66% were U.S. civilians and only one third reported taking malaria chemoprophylaxis [9].

In Europe, international surveillance networks on imported diseases do not specify the number of pregnant women within female malaria cases in the patient characteristics topic [10]. In Spain, there is a lack of epidemiological and clinical data from pregnant women, as this item is not always coded in the communicable diseases' sheet. This is the first description of imported malaria in pregnancy to Spain and one of the few case series in Europe [1,3].

Previous reports on imported malaria have already described the high density of migrant population within the catchment population of both hospitals and the high proportion of immigrants not taking correct prophylaxis [5]. Given that a large proportion of Spain's malaria cases are reported from Madrid, and particularly from the south and eastern districts with annual incidence rates around 7/100,000 population during the last decade, this case series could describe the actual picture of imported malaria in pregnancy in Spain.

Table 2 summarizes the key epidemiological and clinical characteristics from previous case descriptions. Interestingly, though VFRs have been previously identified as a major risk group for imported malaria to Europe, the present and previous case series highlight the fact that, still, VFRs remain a most at risk group during pregnancy and should be better targeted for preventive interventions [11].

**Table 2:** Key clinical and epidemiological findings from three previous studies

	**Subramanian D **[2]	**Westeyn JC **[3]	**Botelho-Nevers E **[1]
Year publication	1992	1995	2005

Institution, country	General hospital, U.S.A	Royal Tropical Institute, The Netherlands	Two maternity wards, France

N° cases (period)	4 (8 months)	10 among 1444 imported malaria cases (9 years)	18 (5 years)

Epidemiological data	3 VFR 1 recently arrived migrant Average age 28 No data on chemoprophylaxis	Not specified 2 adequate chemoprophylaxis	16 VFR 2 national travellers Average age 27 15 no chemoprophylaxis, 3 incomplete

Obstetrical data	1 primigravidae 3 multigravidae	Not specified	4 primigravidae 14 multigravidae

Clinical data			
Presentation	Fever (4/4) Diagnostic delay (2/4)	Not specified	Fever at or 48 hours before presentation (18/18)
Complications	Anaemia (4/4)	Not specified (6/10)	Anaemia (18/18) Severe malaria: Hb < 5 g/dl (2/18) Hypoglycemia (2/18)
Maternal death	1/4	None	None
Foetal outcome	1 congenital malaria (*P.vivax*)	Not specified	1 premature abortion 2 late abortions 1 premature delivery 1 low birth weight

There were neither national travellers nor patients from other endemic areas such as Latin America or Asia, though few malaria cases within this other groups have been reported in both hospitals. The large proportion of sub-Saharan immigrants compared to those of different origin and the fact that tropical areas are an uncommon travel destiny among the national low-middle income population attended at our institutions could explain this finding. Less severe presentations due to *P. vivax *in Latin American pregnant women could also possibly be misdiagnosed. Globally, results from the European Network on Tropical Diseases show that the proportion of imported cases caused by *P. falciparum *in VFR has increased in the last decade. Concomitantly, patient characteristics are different in the other malaria species, where European travellers make up the predominant group. The present and previous studies are consistent with the epidemiological trend in Europe and lead to several questions [10].

First, from the patients' side, are sociocultural and demographic variables influencing on perceived susceptibility and severity of malaria in pregnancy and recognition of malaria and anaemia during pregnancy? Frequently, migrants travelling to their countries of origin mistakenly believe that they retain lifelong immunity and do not seek advice prior to travel nor take appropriate prophylaxis, as was observed in this and previous studies. Also, illness recognition may be hindered and women might confuse malaria symptoms with pregnancy-related symptoms. Socio-cultural aspects on the specific topic of malaria in pregnancy have received little attention in endemic areas [12]. Furthermore, health-seeking behaviour, knowledge, attitudes and perceptions towards pregnancy and pregnancy-related risks in the field of malaria within the immigrant population are under-researched in non-endemic countries. Health promotion to this vulnerable group needs to be better addressed.

From the health system's side, is there availability and accessibility of health advice in antenatal care clinics and primary care regarding malaria? Health staff attending pregnant immigrant women is crucial in recognizing the potential risk of travel and in considering malaria when a diagnostic work-up is carried out. A large proportion of immigrant patients seek care in general hospitals, situated near their homes, where a tropical diseases department is not always available. Pregnant women are usually attended by obstetricians and general practitioners, who might not be familiarized with this type of malaria. In this study, pregnancies were classified as high risk because of co-morbid conditions and not because of malaria exposure risk. Only in one case exposure was considered, and this was because the patient had had fever during her stay. Awareness of this issue should be raised among physicians, midwives and health policy makers.

Regarding clinical presentation, in malaria, nature of effects depends on the patient's background premunition. In pregnancy, parity-specific immunity is crucial and little is known about loss of this specific immunity years after leaving an endemic zone.

Among the sixteen symptomatic patients, symptoms were non-specific with a predominance of fever and the median diagnostic delay was 7 days. Previous series have attributed delays to misdiagnoses with common illnesses due to unfamiliarity with the disease [2]. The two symptomatic though non-febrile patients referring asthenia had moderate anaemia at diagnosis, low parasitaemia and presented the longest delay in diagnosis. One was a recently arrived immigrant and the other had been in Spain for five years, suggesting the persistence of low level immunity able to restrict parasitaemia. On the other hand, an inappropriate health-seeking behaviour due to unperceived pregnancy-related risk and the perception of symptoms as "normal" pregnancy symptoms might contribute to diagnostic delay.

Similarly, absence of symptoms in three patients might have been due to some degree of pre-existing immunity retained during pregnancy, though time living in Spain ranged widely from four months to six years. Many pregnant women living in high transmission areas are known to remain asymptomatic. It is noteworthy that two asymptomatic patients had anaemia, one of which was severe. In fact, a study in Mozambique found no evidence of association between signs and symptoms suggestive of malaria and the presence of microscopically detected peripheral malaria, but anaemia was the only sign associated with sub-microscopic parasitaemia detected exclusively by PCR, suggesting that low density asymptomatic infections may increase the risk of anaemia [13].

Anaemia (WHO definition: haemoglobin ≤ 11 g/dl) is one of the world's leading causes of disability, affecting nearly half of all pregnant women in the world: 52% in developing countries, 23% in the developed world [14]. A strong association between severe anaemia and maternal mortality has been found and associated perinatal outcomes have been documented [15]. Reduction of anaemia is thus a key component of safe motherhood.

One of the most common causes of anaemia is malaria. In the study on sub-microscopic *P. falciparum *malaria in Mozambican pregnant women, risk of maternal anaemia was higher in PCR positive women than in PCR negative (OR 1.92 CI 1.09-3.36) [13]. WHO recommends anti-malarial treatment in pregnant women with severe anaemia living in areas where the risk of *P. falciparum *infections is high even in the absence of parasitaemia [16]. Anaemia was the most common maternal consequence of malaria in the present and previous studies, even in otherwise asymptomatic patients. Three patients presented severe anaemia requiring blood transfusion in addition to anti-malarials. The incidence of maternal anaemia is higher in first pregnancies in malarious areas as a consequence of increased parasite prevalence and severity of infection. The only primigravid patient came to hospital at the end of pregnancy and referred no other symptoms apart from those attributed to labour. But, contrarily, she had severe anaemia and it was this finding that finally led to the diagnosis of malaria. It is, therefore, important to identify anaemic patients and to consider malaria as a possible underlying cause in any pregnant woman with exposure history in order to treat them adequately.

Other complications observed in the present and in previous reports were hypoglycaemia and splenomegaly. Similarly to the French series, there were no maternal deaths nor other complications apart from those mentioned above, suggesting that probably, this mainly multigravidae group of women retained a certain level of parity-specific immunity years after leaving endemic areas. But adverse foetal outcomes in both series did occur in the form of abortions and preterm delivery of a LBW neonate. One fatal maternal case was described in the U.S. report: this patient was primigravidae, diagnosis was delayed and she progressed towards severe complications (hypoglycaemia and respiratory distress) [2].

Thus, globally, clinical suspicion in a non-endemic area such as Spain may be challenging due to the unfamiliarity with the disease and with the link between anaemia and malaria and the low specificity of clinical complaints and signs. Prompt diagnosis and treatment will depend on illness recognition and the women's health-seeking behaviour based on her perceived susceptibility to malaria.

During pregnancy, asymptomatic low parasite densities and parasites sequestered in the placenta are harmful to the mother and foetus, and the sensitivity of microsocopy might be insufficient in these cases. Moreover, parasitaemia can fluctuate and be kept under level of detection by acquired immunity or self-medication, particularly late in pregnancy, and recrudescences can occur later than in non-pregnant patients [13]. This can make diagnosis difficult to less experienced and trained microscopists in non-endemic areas.

Patients received treatment following current guidelines and depending on availability of drugs. Following treatment it is necessary to reliably confirm infection and clearance of parasites. In this study, follow up of nearly 40% of patients was not complete to guarantee parasite clearance. But, assessment of the efficacy of treatment is complicated. Except when the treatment occurs near delivery, to know whether current regimens are able to clear placental parasites requires sensitive diagnostic tools. PCR allows the identification of parasitaemia levels below the threshold of microscopy and can be useful in predicting adverse outcomes in pregnancy [17].

Acquired anti-malarial immunity depends not only on the intensity of transmission and the number of previous pregnancies, but also on the presence of conditions that decrease immunity such as HIV infection. HIV infection restricts the development of parity-specific immunity, has an additive effect on LBW and anaemia and has been shown to cause more clinical malaria and higher parasitaemia in perennial transmission areas, and higher rates of severe episodes and mortality in areas with seasonal transmission. Regarding the role of placental malaria in HIV mother to child transmission (MTCT), results are controversial. Both maternal HIV RNA viral load and anaemia are independent risk factors for MTCT [18]. There were three patients with HIV coinfection. Surprisingly, two of them presented no symptoms but did have anaemia. All classified as CDC category A, stages 1 and 2 which may have resulted in an unremarkable clinical progress, without adverse foetal outcomes. Other than the significant prevalence of coinfection in this series (15%), no other conclusions can be drawn due to the small number of patients.

## Conclusions

This is a small-sample size, retrospective, hospital-based descriptive study with the limitations that pertain to the nature of the study design. Nevertheless, these findings highlight that falciparum malaria needs to be considered as a differential diagnosis when dealing with pregnant women who might have been exposed in endemic areas, particularly sub-Saharan VFRs, even in the absence of symptoms, and should be especially considered in patients with unexplained anaemia.

Uptake of chemoprophylaxis is still low in immigrant travellers. Hence, tailoring health messages to immigrant groups is a priority and access to information on prevention, chemoprophylaxis and health-seeking attitudes should be made available to immigrants in general hospitals.

## Competing interests

The authors declare that they have no competing interests.

## Authors' contributions

BCJ, JMRG and GRM designed the study. All authors complied data and contributed to the bibliographic research. BCJ, JMRG, GRM created the database and interpreted the results. BCJ performed the statistical analysis and drafted the article and JMRG, GRM, PCT and JCG revised it critically. All authors approved the final manuscript.
